# Comparison Between First Line Target Therapy and Immunotherapy in Different Prognostic Categories of BRAF Mutant Metastatic Melanoma Patients: An Italian Melanoma Intergroup Study

**DOI:** 10.3389/fonc.2022.917999

**Published:** 2022-08-15

**Authors:** Riccardo Marconcini, Paolo Fava, Amedeo Nuzzo, Simona Manacorda, Marco Ferrari, Francesco De Rosa, Michele De Tursi, Enrica Teresa Tanda, Francesca Consoli, Alessandro Minisini, Nicola Pimpinelli, Francesca Morgese, Melissa Bersanelli, Marco Tucci, Maristella Saponara, Alessandro Parisi, Marcella Ocelli, Serena Bazzurri, Giulia Massaro, Riccardo Morganti, Isabella Ciardetti, Ignazio Stanganelli

**Affiliations:** ^1^ Unit of Medical Oncology 2, Azienda Ospedaliero-Universitaria Pisana, Pisa, Italy; ^2^ Struttura Complessa (S.C.) Dermatologia Azienda Ospedaliero Universitaria (AOU) Città della Salute e della Scienza di Torino, Torino, Italy; ^3^ Istituto di Ricovero e Cura a Carattere Scientifico (IRCCS) Istituto Romagnolo per lo studio dei Tumori “Dino Amadori”, Meldola, Italy; ^4^ Dipartimento di Tecnologie Innovative in Medicina & Odontoiatria Sezione di Oncologia Università G. D’Annunzio Chieti-Pescara, Chieti-Pescara, Italy; ^5^ Istituto di Ricovero e Cura a Carattere Scientifico (IRCCS) Ospedale Policlinico San Martino, Genoa, Italy; ^6^ Genetics of Rare Cancers, Department of Internal Medicine and Medical Specialties (DIMI), University of Genoa, Genoa, Italy; ^7^ Unitá Operativa (U.O.) Oncologia Medica, Azienda Socio Sanitaria Territoriale (ASST) Spedali Civili, Brescia, Italy; ^8^ Dipartimento di Oncologia Azienda Sanitaria Universitaria del Friuli Centrale P.le Santa Maria (SM) della Misericordia, Udine, Italy; ^9^ Dipartimento Di Scienze Della Salute (DSS), Sezione Dermatologia, Università di Firenze, Melanoma & Skin Cancer Unit Area Vasta Centro, Firenze, Italy; ^10^ Clinica Oncologica, Azienda Ospedaliero-Universitaria Ospedali Riuniti Umberto I, G.M. Lancisi, G. Salesi di Ancona, Ancona, Italy; ^11^ Unità Operativa di Oncologia Medica, Azienda Ospedaliero-Universitaria di Parma e Dipartimento di Medicina e Chirurgia, Università degli Studi di Parma, Parma, Italy; ^12^ Medical Oncology Unit, Department of interdisciplinary Medicine (DIM), University of Bari ‘Aldo Moro’, Bari, Italy; ^13^ Istituto Europeo Oncologico—Milano, Milano, Italy; ^14^ Medical Oncology, St. Salvatore Hospital, L’Aquila, Italy; ^15^ Oncologia Medica Cuneo, Cuneo, Italy; ^16^ Section of Statistics—University Hospital of Pisa, Pisa, Italy; ^17^ Skin Cancer Unit, Scientific Institute of Romagna for the Study of Cancer, Istituto di Ricovero e Cura a Carattere Scientifico (IRCCS) Istituto per La Ricerca Scientifica e Tecnologica (IRST), Meldola, Italy; ^18^ Department of Dermatology, University of Parma, Parma, Italy

**Keywords:** melanoma, BRAF mutated, targeted therapy, immunotherapy, first line

## Abstract

**Background:**

BRAF and MEK inhibitors target therapies (TT) and AntiPD1 immunotherapies (IT) are available first-line treatments for BRAF v600 mutant metastatic melanoma patients. ECOG PS (E), baseline LDH (L), and baseline number of metastatic sites (N) are well-known clinical prognostic markers that identify different prognostic categories of patients. Direct comparison between first-line TT and IT in different prognostic categories could help in first line treatment decision.

**Methods:**

This is a retrospective analysis conducted in 14 Italian centers on about 454 metastatic melanoma patients, divided in 3 groups: group A—patients with E = 0, L within normal range, and N less than 3; group B—patients not included in group A or C; group C—patients with E > 0, L over the normal range, and N more than 3. For each prognostic group, we compared TT and IT in terms of progression free survival (PFS), overall survival (OS), and disease control rate (DCR).

**Results:**

In group A, results in 140 TT and 36 IT-treated patients were, respectively, median PFS 35.5 vs 11.6 months (HR (95% CI) 1.949 (1.180–3.217) *p* value 0.009); median OS not reached vs 55 months (HR (95% CI) 1.195 (0.602–2.373) *p* value 0.610); DCR 99% vs 75% *p* value <0.001). In group B, results in 196 TT and 38 IT-treated patients were, respectively, median PFS 11.5 vs 5 months (HR 1.535 (1.036–2.275) *p* value 0.033); median OS 19 vs 20 months (HR 0.886 (0.546–1.437) *p* value 0.623); DCR 85% vs 47% *p* value <0.001). In group C, results in 41 TT and 3 IT-treated patients were, respectively, median PFS 6.4 vs 1.8 months (HR 4.860 (1.399–16) *p* value 0.013); median OS 9 vs 5 months (HR 3.443 (0.991–11.9) *p* value 0.052); DCR 66% vs 33% *p* value 0.612).

**Conclusions:**

In good prognosis, group A—TT showed statistically significant better PFS than IT, also in a long-term period, suggesting that TT can be a good first line option for this patient category. It is only in group B that we observed a crossing of the survival curves after the 3rd year of observation in favor of IT. Few patients were enrolled in group C, so few conclusions can be made on it.

## Introduction

Worldwide, it is estimated that in the last decade cutaneous melanoma has reached 100,000 new cases per year: an increase of about 15% compared to the previous decade. BRAF is mutated in about 50% of cutaneous melanomas (1) and in patients with BRAF-mutated metastatic cutaneous melanoma multiple therapeutic options are currently available: BRAF-MEK inhibitors targeted therapies, AntiPD1, and AntiCTLA4 immune checkpoint inhibitors (2, 3).

The prognosis of patients with BRAF-mutated melanoma has greatly improved in the last 10 years thanks to the advent of these new drugs. To date, BRAF-MEK inhibitors targeted therapies available are Vemurafenib and Cobimetinib, Dabrafenib and Trametinib, and Encorafenib and Binimetinib, respectively, investigated in the Cobrim (4), Combi-d and Combi-v studies (5, 6), and Columbus trials (7); in these studies, median OS found for each combination were respectively 22.3 months, 25.6 months, and 33.6 months, while in a recent pooled analysis of studies involving Dabrafenib Trametinib, the 5-year survival was 34%.

Regarding immunotherapy, in the Checkmate 067 study Ipilimumab, Nivolumab and the combination of these two inhibitory immune checkpoints demonstrated a 5-year live patient rate of 26, 44, 52%, respectively (8). Despite the benefit observed with both targeted therapy or immunotherapy strategies, some patients develop unpredictable innate or acquired resistance to the treatment administered. To date, there is a debate about which is the most appropriate first-line treatment choice in BRAF-mutated metastatic melanoma. Currently, phase III studies are trying to answer this question such as the Dreamseq study (9), of which some preliminary data have been recently presented, and the ongoing Secombit study (10).

In a previous *post hoc* analyses of both immunotherapy and target therapy, it emerged that some clinical parameters such as ECOG Performance Status (PS), basal LDH, and number of metastatic sites are able to identify different prognostic groups in which innovative therapies perform differently (11–13). In order to identify the best treatment choice in BRAF-mutated metastatic melanoma patients, we conduct a comparison between treatments. Pending the results of ongoing randomized studies, real word data can help to answer the question about the optimal strategy to choose as first line treatment in BRAF mutated stage IV melanoma patients.

Up to the late 2021, in Italy, the Ipilimumab Nivolumab combination was approved but not reimbursed from the National Health System, thus, was not available outside Clinical Trials. We conducted a retrospective study in 14 Italian centers, with the aim of comparing first line use of BRAF-MEK inhibitors or AntiPD1 immunotherapy, in different prognostic groups identified by LDH, ECOG PS, and number of metastatic sites, to verify the best treatment within each prognostic category of patients.

## Material and Methods

We conducted an observational multicenter retrospective study involving 14 Italian Centers. Eligible patients were 18 years of age and older, had a diagnosis of BRAF V600-mutated metastatic melanoma, and had received first-line systemic treatment for metastatic melanoma with BRAF-MEK inhibitors target therapy or AntiPD1 immunotherapy. The presence of brain metastases was an exclusion criterion. Patients were divided into three different prognostic risk categories: group A (patients with ECOG PS 0, LDH within the normal range, and number of metastatic sites less than 3), group B (patients not included in group A or C), and group C (patients with ECOG PS greater than 0, LDH over the normal range, and number of metastatic sites greater than 3). For each prognostic category, we compared target therapy and immunotherapy in terms of progression-free survival (PFS), overall survival (OS), objective response rate (ORR), and disease control rate (DCR).

### Statistical Analysis

Categorical data were described by absolute and relative (%) frequency, continuous data by mean and standard deviation. Type therapy was compared with qualitative and quantitative factors using chi-square test or *z* test for two proportions (when appropriate) and *t* test for independent sample (two-tailed), respectively. PFS and OS were analyzed, survival curves were calculated using Kaplan–Meier method, and log-rank test was applied to evaluate differences between curves. Influence of the therapy on the survival, stratified for risk, was also analyzed and hazard ratio (HR) with 95% CI was indicated. Significance was fixed at 0.05 and all analyses were carried out with SPSS v. 27 technology.

## Results

### Patients Population

A total of 454 patients were enrolled. Baseline characteristics of the population are summarized in **Table 1**. Within the total population, 377 (83%) patients received target therapies and 77 (17%) immunotherapies. Most patients were male (59.7%) and had an ECOG PS of 0 (76%). The mean age was 61 0years. Baseline LDH was within the normal range in 265 patients (58.4%) and 303 (66.7%) patients had less than 3 metastatic sites. The mean follow-up was 38.4 months.

**Table 1:** Population characteristics (*n* = 454). Statistics: frequency (%) or media (ds).

Characteristic	Total	Target therapies	Immunotherapy	*p* value
**Gender**				0.617
Male	271 (59.7)	227	44	
Female	183 (40.3)	150	33	
ECOG performance status (baseline)				0.005
0	345 (76)	277	68	
1	109 (24)	100	9	
**LDH value baseline**				0.989
Normal	265 (58.4)	220	45	
>Normal value	189 (41.6)	157	32	
**Number of metastatic sites**				0.221
<3	303 (66.7)	247	56	
>3	151 (33.3)	130	21	
**Prognostic group**				0.090
Group A (better prognosis)	176 (38.8)	140	36	
Group B (intermediate prognosis)	234 (51.5)	196	38	
Group C (worse prognosis)	44 (9.7)	41	3	
**Age**	61 (15)	61 (15)	59 (14)	0.422
**Time between primary melanoma removal and metastasis diagnosis**	41 (53)	41 (53)	38.2 (56)	0.665
**NLR (neutrophils lymphocytes ratio)**	3.4 (3.3)	3.4 (3.2)	3.1 (2.3)	0.526

### ORR and DCR

Within each group, treatment with target therapy showed the greatest benefit in terms of ORR and DCR (**Tables 2A** and **2B**).

**Table 2A:** Response rate.

Prognostic group	Therapy	CR	PR	SD	PD	Tot
**Group A**	Target therapy	50	72	15	5	142
	Immunotherapy	5	9	10	9	33
**Group B**	Target therapy	31	104	26	34	195
	Immunotherapy	3	9	6	21	39
**Group C**	Target therapy	1	19	7	15	42
	Immunotherapy	0	0	1	2	3

**Table 2B:** ORR and DCR: comparison between target and immunotherapies.

Prognostic group	Therapy	ORR (%)	*p* value	DCR (%)	*p* value
**Group A (better prognosis)**	Target therapy	87.8	<0.001	98.6	<0.001
	Immunotherapy	43.8		75	
**Group B (intermediate prognosis)**	Target therapy	71	<0.001	84.7	<0.001
	Immunotherapy	31.6		47.4	
**Group C (worse prognosis)**	Target therapy	48.8	0.299	65.8	0.612
	Immunotherapy	0		33.3	

In group A (best prognosis), treatment with target therapy shows ORR (87.8% vs 43.8%, *p* < 0.001) and DCR (98.6% vs 75%, *p* < 0.001) compared to immunotherapy.

In group B (intermediate prognosis), treatment with target therapy was also more beneficial than immunotherapy for ORR (71% vs 31.6%, *p* < 0.001) and DCR (84.7% vs 47.4%, *p* < 0.001).

In group C (worst prognosis), treatment with target therapy performed better than immunotherapy for ORR (48.8% *vs* 0%, *p* < 0.299) and DCR (65.8% vs 33.3%, *p* < 0.612), but due to the limited number of patients treated with immunotherapy in this prognostic group, this analysis did not reach statistical significance and did not allow any definitive conclusions to be drawn.

### PFS and OS

**Figure 1:**
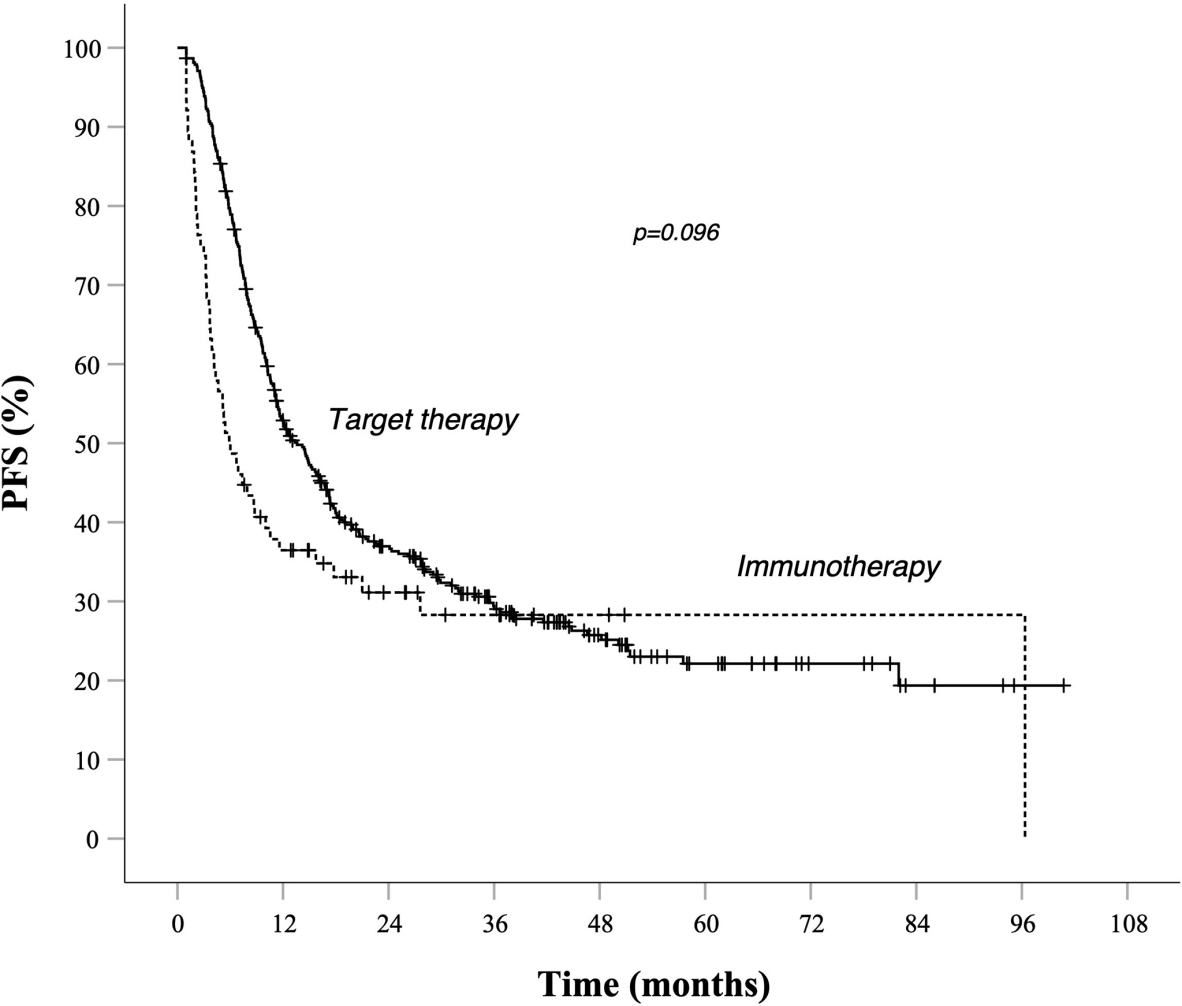
PFS in total population Kaplan–Meier curves. Target therapy: median PFS (95% CI) 13.6 (11.1–16.1) months. AntiPd1 immunotherapy: median PFS (95% CI) 5.9 (3.5–8.3) months—*p* value 0.096. Survival rate at 1, 2, 3, and 5 years data are specified in **Tables 3** and **4**. —*Continuous line*: patients treated with BRAF-MEK inhibitors target therapies. *Dotted line*: patients treated with AntiPD1 Immunotherapy—HR is not reported in case of crossing curves because not statistically relevant.

**Figure 3:**
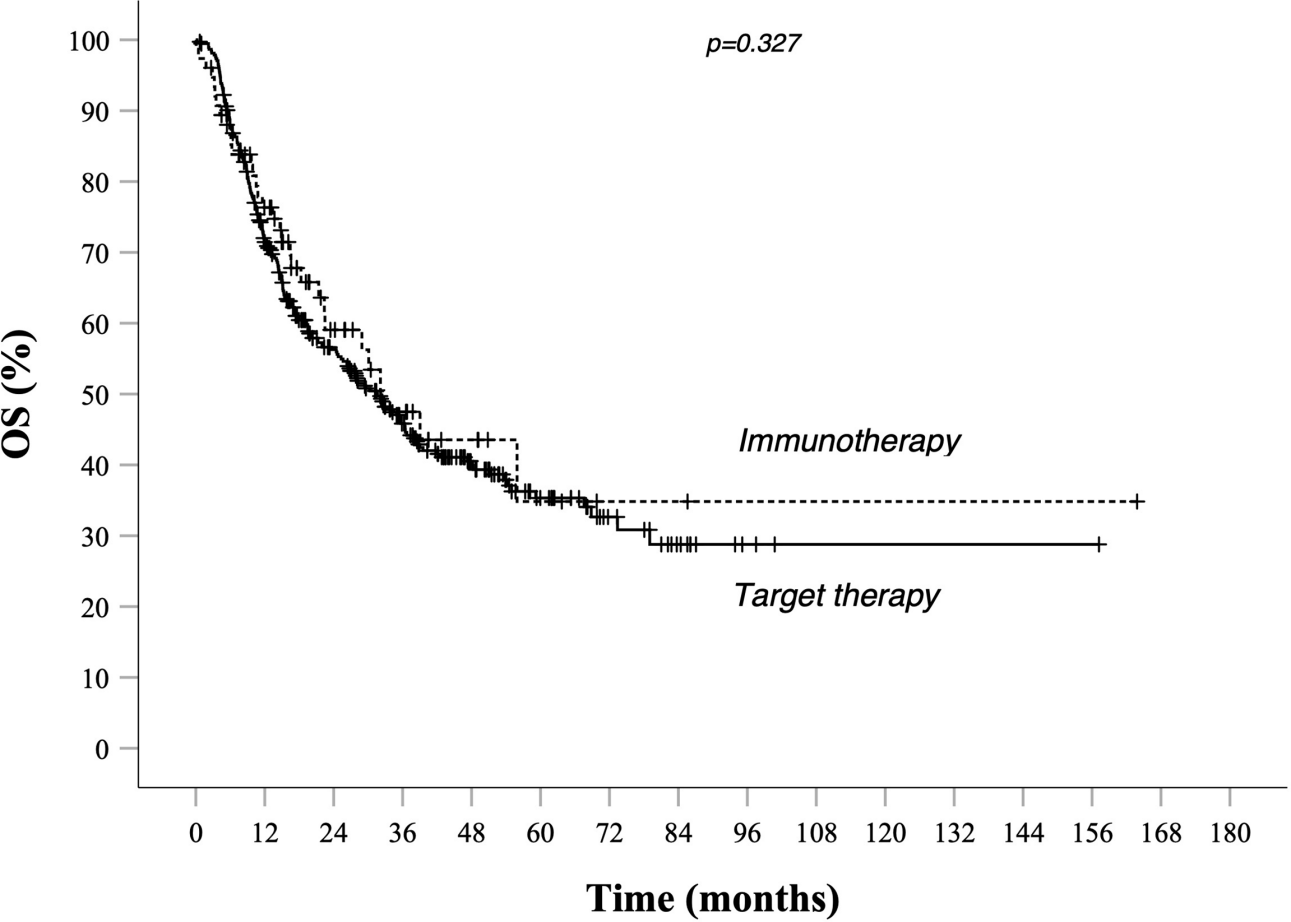
OS in total population Kaplan–Meier curves. BRAF-MEK inhibitor target therapy median OS (95% CI) 31.6 (25.5–37.7)—AntiPd1 Immunotherapy median OS (95% CI): 32.7 (21.3–44.2) *p* value 0.327. Survival rate at 1, 2, 3, and 5 years data are specified in **Tables 3** and **4**. *Continuous line*: patients treated with BRAF-MEK inhibitors target therapies. *Dotted line*: patients treated with AntiPD1 Immunotherapy—HR is not reported in case of crossing curves because not statistically relevant.

Survival rates at different time points stratified by prognostic risk groups are summarized in **Table 3**. As illustrated in **Table 4**, evaluation for OS showed that median OS for total population was similar in target therapy and immunotherapy group, respectively, 31.6 (25.5–37.7) and 32.7 (21.3–44.2) months (**Figure 3**). On the other hand, in the total population, PFS in total population PFS was not statistically different between patients treated with AntiPD1 immunotherapy or BRAF-MEK inhibitors. As shown in **Figure 1**, PFS Kaplan–Meier curves cross each other during observation. (**Figure 1**).

**Table 3:** Survival rate at 1, 2, 3, and 5 years stratified by prognostic risk groups. nr: not reached.

Patients group	Time	Target therapy		Immunotherapy	
		PFS (%)	OS (%)	PFS (%)	OS (%)
**Group A**	1 year	70	88	48	80
	2 year	57	80	43	77
	3 year	48	65	37	63
	5 year	43	55	nr	43
**Group B**	1 year	40	64	29	75
	2 year	30	48	23	48
	3 year	22	36	23	37
	5 year	12	27	23	30
**Group C**	1 year	18	28	nr	nr
	2 year	nr	10	nr	nr
	3 year	nr	5	nr	nr
	5 year	nr	nr	nr	nr

**Table 4:** Median survival times and 95% CI. nr: not reached. (HR, *p* value relative to comparison between target therapy and immunotherapy are shown in **Figures 1** and **2**).

Patients group	Target therapy		Immunotherapy	
	Median PFS (95% CI)	Median OS (95% CI)	Median PFS (95% CI)	Median OS (95% CI)
**Tot**	13.6 (11.1–16.1)	31.6 (25.5–37.7)	5.9 (3.5–8.3)	32.7 (21.3–44.2)
**Group A**	35.5 (21.7–49.3)	nr	11.6 (1–24.9)	55.9 (15.6–96.2)
**Group B**	10.5 (9.2–11.9)	19.6 (12.4–26.8)	3.7 (2.5–5)	22.4 (12.6–32.4)
**Group C**	6.4 (5–7.7)	9.1 (6.1–12)	1.7 (1–2.5)	5.4 (2.4–8.4)

**Figure 2:**
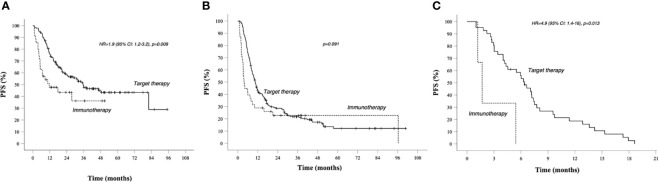
PFS in prognostic groups A, B, C, Kaplan–Meier curves. **(A)** PFS in Group A (better prognosis patients). **(B)** PFS in Group B (intermediate prognosis patients). **(C)** PFS in worse prognosis patients. Median survival times and 95% CI. Survival rate at 1, 2, 3, and 5 years data are specified in **Tables 3** and **4**. *Continuous line*: patients treated with BRAF-MEK inhibitors target therapies. *Dotted line*: patients treated with AntiPD1 Immunotherapy—HR are reported only in case of significant difference between curves; HR is not reported in case of crossing curves because not statistically relevant.

**Figure 4:**
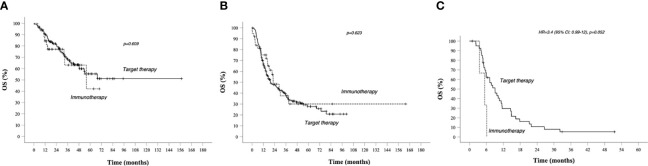
OS in prognostic groups A, B, C, and Kaplan–Meier curves. **(A)** OS in Group A (better prognosis patients). **(B)** OS in Group B (intermediate prognosis patients); **(C)** OS in worse prognosis patients. Median survival times and 95% CI. Survival rate at 1, 2, 3, and 5 years data are specified in **Tables 3** and **4**. *Continuous line*: patients treated with BRAF-MEK inhibitors target therapies. *Dotted line*: patients treated with AntiPD1 Immunotherapy—HR are reported only in case of significant difference between curves; HR is not reported in case of crossing curves because not statistically relevant.

In group A, target therapy showed significant better PFS than immunotherapy, also in the long-term period, with a median PFS of 35.5 vs 11.6 months, respectively (HR 1.9, 95% CI 1.2–3.2, *p* = 0.009; **Figure 2A**). In this group, the OS for immunotherapy was 55.9 months (15.6–96.2), and for the target therapy, it was still not reached (**Figure 4A**).

In group B, we observed that median PFS for target therapy and immunotherapy was, respectively, 10.5 (9.2–11.9) and 3.7 (2.5–5) months, HR 1.535 (95% CI 1.036–2.275, *p* = 0.033). However, these results are not statistically relevant due to the crossing of curves. As a matter of fact, target therapy showed a better PFS than immunotherapy during the first 2 years of observation (PFS at 2 years: 30% *vs* 23%), and after the third year, we saw a change of the trend (PFS at 5 years: 12% *vs* 23%; **Figure 2B**).

As shown in **Table 3**, patients treated with immunotherapy achieved better absolute OS rates than target therapy at different time points. However, looking at median OS, this analysis did not reach the statistical significance: median OS for target therapy and immunotherapy was, respectively, 19.6 (12.4–26.8) and 22.4 (12.6–32.4) months, HR 0.886 (95% CI 0.546–1.437, *p* = 0.623; **Figure 4B**).

In group C, target therapy showed greater efficacy than immunotherapy, with a median PFS of 6.4 (5–7.7) and 5.4 (2.4–8.4) months, respectively (HR 4.9, 95% CI 1.4–16, *p* = 0.013; **Figure 2C**). Instead, median OS was 9.1 (6.1–12) and 5.4 (2.4–8.4), respectively (HR 3.4, 95% CI 0.99–12, *p* = 0.052; **Figure 4C**). However, due to the few patients enrolled in group C, we cannot make any final conclusion.

### Safety Profile

Adverse events of treatments are reported in **Table 5**. The toxicity data we found in our series are in line with those reported in the literature for the BRAF-MEK inhibitors and for AntiPD1.

**Table 5:** Treatments adverse event.

Treatment		BRAF+MEK Inhibitors				Immunotherapy			
Event CTCAE grade		1	2	3	4	1	2	3	4
Skin toxicity	Absolute court	70	68	60	4	14	10	12	0
	%	17.37	16.87	14.89	0.99	17.07	12.20	14.63	0.00
Pirexia	Absolute court	92	100	27	0	3	6	3	0
	%	22.83	24.81	6.70	0.00	3,66	7.32	3,66	0.00
Nausea/Vomiting	Absolute court	33	24	15	0	1	8	0	0
	%	8.19	5.96	3.72	0.00	1.22	9.76	0.00	0.00
Diarrhea	Absolute court	31	26	15	0	5	6	3	0
	%	7.69	6.45	3.72	0.00	6.10	7.32	3.66	0.00
Tyroid function alteration	Absolute court	0	4	0	0	6	8	0	0
	%	0.00	0.99	0.00	0.00	7.32	9.76	0.00	0.00
Surrenalic gland alteration	Absolute court	0	0	0	0	0	2	3	0
	%	0.00	0.00	0.00	0.00	0.00	2.44	3.66	0.00
Hypofisitis	Absolute court	0	0	0	0	0	2	0	0
	%	0.00	0.00	0.00	0.00	0.00	2.44	0.00	0.00
Lung toxicity	Absolute court	0	2	0	0	2	3	0	4
	%	0.00	0.50	0.00	0	2.44	5.7	0	4.88
Cardiologic toxicity	Absolute court	13	18	27	0	0	0	0	0
	%	3.23	4.47	6.70	0.00	0.00	0.00	0.00	0.00
Increase in transaminasis	Absolute court	12	14	18	0	2	0	4	0
	%	2.98	3.47	4.47	0.00	2.44	0.00	4.88	0.00
Kidney function reduction	Absolute court	5	2	0	0	1	0	0	0
	%	1.34	0.50	0.00	0.00	1.22	0.00	0.00	0.00
Arthralgia	Absolute court	11	6	6	0	3	3	2	0
	%	2.73	1.49	1.49	0.00	3.66	3.66	2.44	0.00
Neurologic toxicity	Absolute court	2	4	0	0	0	0	0	0
	%	0.50	0.99	0.00	0.00	0.00	0.00	0.00	0.00
Neutropenia	Absolute court	0	14	9	0	0	0	0	0
	%	0.00	3.47	2.23	0.00	0.00	0.00	0.00	0.00
Anemia	Absolute court	1	0	3	0	0	0	0	0
	%	0.25	0.00	0.74	0.00	0.00	0.00	0.00	0.00
Astenia	Absolute court	15	14	0	0	1	2	0	0
	%	3.72	3.47	0.00	0.00	1.22	2.44	3.66	0.00
Pancreatitis	Absolute court	0	2	0	0	0	2	3	0
	%	0.00	0.50	0.00	0.00	0.00	2.44	3.66	0.00
CPK incremental level	Absolute court	3	4	3	0	0	0	0	0
	%	0.74	0.99	0.74	0.00	0.00	0.00	0.00	0.00
Uveitis	Absolute court	0	4	3	0	0	0	0	0
	%	0.00	0.99	0.74	0.00	0.00	0.00	0.00	0.00
Visus reduction	Absolute court	0	4	0	0	0	0	0	0
	%	0.00	0.99	0.00	0.00	0.00	0.00	0.00	0.00
Dysgeusia	Absolute court	5	4	0	0	0	0	0	0
	%	1.24	0.99	0.00	0.00	0.00	0.00	0.00	0.00
Treatment related Adverse Events leading to discontinuation	Absolute court	14				7			
	%	3.47				8.54			

## Discussion

In the modern era, there is a debate about which is the best first line treatment between targeted therapies and immunotherapy in stage IV BRAF-mutated melanoma patients (14, 15). As part of both therapeutic strategies, various *post hoc* analyzes carried out in multiple studies have shown how some simple clinical laboratory parameters constitute prognostic factors capable of identifying groups of patients with different life perspectives and with different therapeutic performances.

Several *post hoc* analyses showed that some simple features, such as baseline ECOG PS, LDH value, and number of metastatic sites, can be used to successfully identify patients with different clinical outcomes and prognoses. This work aims to better clarify which treatment between BRAF-MEK inhibitors and AntiPD1 performed best when used as the first line therapy in each group of patients with different prognoses (good, intermediate, and poor prognosis groups) (16, 17). Thus, the comparison appears less influenced by the biases inherent to the prognosis of the patients and closer to the real life setting (18). Patients with brain metastases were excluded from the present study due to the extremely poor prognosis and the different treatment options of this subgroup (18–20).

As previously described, the group of patients with good prognosis was defined by the coexistence of the following facts: ECOG PS 0, normal LDH, and less than three metastatic sites. In this group (group A), the target therapies showed a particular activity with an ORR significantly higher than that observed in the patients treated with AntiPD1 (87.8% *vs* 43.8%, *p* < 0.001). In addition, 35% of patients achieved a complete response with the administration of BRAF-MEK inhibitors. In this group, we observe a statistically significant advantage in terms of PFS in favor of the targets on AntiPD1 (HR 1.9 95% CI 1.2–3.2, *p* = 0.009) and a substantial overlap of the curves in terms of OS with rates of comparable survival over a long observation period. It can therefore be observed that in this group with a particularly favorable prognosis, both AntiPD1 and the target therapies have a high percentage of alive patients, with an advantage in terms of activity and PFS for the target therapies, which therefore seem to be an effective alternative in this setting (22).

The worst prognosis group (group C) was defined by the coexistence of the following baseline factors: ECGPS > 0, LDH > normal, and more than three metastatic sites. The clinical goal in this group is to obtain immediate clinical benefit, while the chances of achieving long survival are reduced. Both BRAF-MEK inhibitors and AntiPD1 treatment reduced ORR in this subgroup of patients. Only 3 patients of this prognostic category were treated with AntiPD1 and it is not possible to make a definitive comparison between the two therapeutic strategies. However, the fact that clinicians in almost all cases preferred to use target therapies indicates a widespread preference for this treatment in this area. The known shrinking capacity of target therapies seems to make this strategy particularly active and preferred by clinicians in this category with poorer prognosis.

In the intermediate prognosis group (group B), defined by patients not included in the two previous groups, and with intermediate characteristics with respect to the prognostic factors considered, BRAF-MEK inhibitors show greater activity than AntiPD1 (ORR 71% *vs* 31, 6%, *p* < 0.001); in terms of PFS and OS, we observed the phenomenon of the intersection of Kaplan–Meier curves. Initially, in the first months of observation, the target therapies have a majority of patients live and not progressed compared to AntiPD1, but in prolonged observation, the percentage of alive and progression-free patients is higher in the AntiPD1 treated group.

Our study has some limitations. First, the possible biases related to the retrospective design of the study. However, it should be noted that the number of patients enrolled is considerable. Moreover, in our study, patients treated with immunotherapy received AntiPD1 alone (and not the Ipilimumab Nivolumab combination regimen, which has gained particular relevance in recent years). At the time of data collection, Ipilimumab plus Nivolumab combination treatment was not reimbursable in Italy and its availability was limited in the participating centers. However, we believe that AntiPD1 monotherapy remains a central treatment in the management landscape of mutated BRAF melanoma patients and we believe that studies such as this can help frame its right place in the therapeutic decision-making algorithm. At least, we noted that the utilization ratio between targeted therapies and AntiPD1 was approximately 5:1 and this may limit the statistical power of the comparison.

To answer the question of what is the best first-line strategy in patients with BRAF-mutated metastatic melanoma between target therapies and immunotherapy, many phase III studies are ongoing and results are pending [e.g., Secombit (10), Dreamseq (9)]. Moreover, in these studies, the immunotherapy administered consisted of the immunocombos Nivolumab plus Ipilimumab, and therefore, the question of our study is not fully comparable to these clinical trials. At the moment, evidence from the real world such as our study can be of help in framing the problem.

Some real word studies in the literature have attempted to answer the question, using alternative statistical methods such as propensity score and coming to the conclusion of recommending the use of AntiPD1 in the first line of treatment (21).Our study takes a different approach: the peculiarity of our study is to perform the comparison of the treatment strategy in the context of different prognostic groups of patients. Probably, the comparison between treatments used on the first line in BRAF mutated metastatic melanoma may not be exhaustive by aggregating data from patients with completely different prognoses. In fact, our three groups of patients showed different activity and efficacy for each therapy applied. The safety profile of BRAF-MEK inhibitors and AntiPD1 immunotherapy in our series is similar to the data reported in the main randomized studies. The discussion on the expected toxicity profile of the BRAF-MEK inhibitor and of the treatment with AntiPD1 could be another element to guide the treatment strategy of physicians in each individual patient, especially in populations with good prognosis where both treatments seem to have the same effectiveness (4–6, 8).

## Conclusion

In conclusion, by dividing patients with BRAF-mutated metastatic melanoma into three prognostic categories based on the baseline assessment of ECOG PS, LDH, and metastatic sites, we noted a different activity and efficacy of both targeted therapies and AntiPD1 monotherapy. In the group with good prognosis, targeted therapies compared to monotherapy with AntiPD1 showed an advantage in activity, PFS, and equivalence in the proportion of patients with long survival; in patients with intermediate prognosis, the majority of long-surviving patients with AntiPD1 appears to be highlighted in absolute terms, while in the poor prognosis group, BRAF-MEK inhibitors are mostly used for their particular activity.

## Data Availability Statement

The raw data supporting the conclusions of this article will be made available by the authors, without undue reservation.

## Ethics Statement

The studies involving human participants were reviewed and approved by Comitato Etico Area Vasta Nord Ovest Sezione Autonoma del Comitato Etico Regionale per la Sperimentazione Clinica- Toscana, Pisa. The patients/participants provided their written informed consent to participate in this study.

## Author Contributions

RMa elaborated the protocol; RMa, AN, and SM elaborated manuscript; RMo performed statistical analysis. All co-author enrolled patients and provided data about enrolled patients, all authors contributed to manuscript elaboration with review and approval of final version of manuscript. JS performed a linguistic review of the manuscript

## Funding

Italian Melanoma Intergroup covers all publication costs related to manuscript publication, but not the execution of this trial.

## Conflict of Interest

RMa, PF, AN, SM, MF, FDR, MDT, ETT, FC, AM, NP, FM, MB, MT, MS, AP, MO, IS declare that they received financial support for congress participation, advisory board, scientific advice from BMS, MSD, Novartis, Pierre fabre, La Roche, Sanofi, Ipsen.

The remaining authors declare that the research was conducted in the absence of any commercial or financial relationships that could be construed as a potential conflict of interest.

## Publisher’s Note

All claims expressed in this article are solely those of the authors and do not necessarily represent those of their affiliated organizations, or those of the publisher, the editors and the reviewers. Any product that may be evaluated in this article, or claim that may be made by its manufacturer, is not guaranteed or endorsed by the publisher.
